# Supplementary material for: Scenario-led modelling of broadleaf forest expansion in Wales
Source: R Soc Open Sci. 2019 May 22;6(5):190026. doi: 10.1098/rsos.190026 (PMC6549994; doi:10.1098/rsos.190026)
Supplement: Revised Land Use Classes & Transitions Sub Models [file rsos190026supp1.docx]

| **Land use Map 2015** | **Land use Map 2007** | **Revised/Harmonized Land use Categories** | **Land use Categories Used in the Model** |
| --- | --- | --- | --- |
| Broadleaved woodland | Broadleaved, mixed and yew woodland | Broadleaved woodland | Broadleaved woodland |
| Coniferous woodland | Coniferous woodland | Coniferous woodland | Coniferous woodland |
| Arable and horticulture | Arable and horticulture | Arable and horticulture | Arable and horticulture |
| Improved grassland | Improved grassland | Improved grassland | Improved grassland |
| Neutral grassland | Rough grassland | Semi-Natural grassland | Semi-Natural grassland |
| Calcareous grassland | Neutral grassland |  |  |
| Acid grassland | Calcareous grassland |  |  |
| Fen, Marsh and Swamp | Acid grassland |  |  |
| Heather | Fen, marsh and swamp | Mountain, Heath, Bog | Mountain, Heath, Bog |
| Heather grassland | Heather |  |  |
| Bog | Heather grassland |  |  |
| Inland rock | Bog |  |  |
| Saltwater | Montane habitats | Water Bodies | ***Category Excluded*** |
| Freshwater | Inland rock |  |  |
| Supra-littoral rock | Saltwater | Coastal | ***Category Excluded*** |
| Supra-littoral sediment | Freshwater |  |  |
| Littoral rock | Supra-littoral rock |  |  |
| Littoral sediment | Supra-littoral sediment |  |  |
| Saltmarsh | Littoral rock |  |  |
| Urban | Littoral sediment | Built Areas | ***Category Excluded*** |
| Suburban | Saltmarsh |  |  |
|  | Urban |  |  |
|  | Sub-Urban |  |  |

**Table S1.** Actual and revised land use classes in the historical land use maps of Wales. Column 1 & 2 show the default legends of the land use maps acquired from Edina Digimap. Column 3 shows the harmonized/revised land use classes. Column 4 shows the finally selected land use classes for the model.

|  | **Sub model** | **Accuracy Rate (%)** |
| --- | --- | --- |
| 1 | Broadleaf Forest to Conifer Forest | 78.56 |
| 2 | Broadleaf Forest to Arable Land | 75.41 |
| 3 | Broadleaf Forest to Improved Grassland | 50.06 |
| 4 | Broadleaf Forest to Semi Natural Grassland | 50.07 |
| 5 | Broadleaf Forest to Mountain, Heath & Bog | 81.85 |
| 6 | Conifer Forest to Broadleaf Forest | 85.47 |
| 7 | Conifer Forest to Improved Grassland | 83.35 |
| 8 | Conifer Forest to Semi Natural Grassland | 70.27 |
| 9 | Arable Land to Broadleaf Forest | 76.81 |
| 10 | Arable Land to Conifer Forest | 93.29 |
| 11 | Arable Land to Improved Grassland | 68.4 |
| 12 | Arable Land to Semi Natural Grassland | 88.7 |
| 13 | Arable Land to Mountain, Heath & Bog | 78.4 |
| 14 | Improved Grassland to Broadleaf Forest | 70.35 |
| 15 | Improved Grassland to Conifer Forest | 80.02 |
| 16 | Improved Grassland to Arable Land | 69.3 |
| 17 | Improved Grassland to Semi Natural Grassland | 88.27 |
| 18 | Improved Grassland to Mountain, Heath & Bog | 75.74 |
| 19 | Semi Natural Grassland to Broadleaf Forest | 91.81 |
| 20 | Semi Natural Grassland to Conifer Forest | 72.28 |
| 21 | Semi Natural Grassland to Arable Land | 95.94 |
| 22 | Semi Natural Grassland to Improved Grassland | 89.33 |
| 23 | Semi Natural Grassland to Mountain, Heath & Bog | 68.87 |
| 24 | Mountain Heath & Bog to Broadleaf Forest | 93.75 |
| 25 | Mountain Heath & Bog to Conifer Forest | 78.88 |
| 26 | Mountain, Heath & Bog to Arable Land | 93.91 |
| 27 | Mountain, Heath & Bog to Improved Grassland | 92.74 |
| 28 | Mountain, Heath & Bog to Semi Natural Grassland | 65.13 |

**Table S2.** Transition sub-models in the study. Accuracy rate determines the precision with which the sub models were trained and validated.
