# Supplementary material for: Scenario-led modelling of broadleaf forest expansion in Wales
Source: R Soc Open Sci. 2019 May 22;6(5):190026. doi: 10.1098/rsos.190026 (PMC6549994; doi:10.1098/rsos.190026)
Supplement: Contribution to net change, Historical LULC transitions and transitions potential maps [file rsos190026supp2.docx]

**
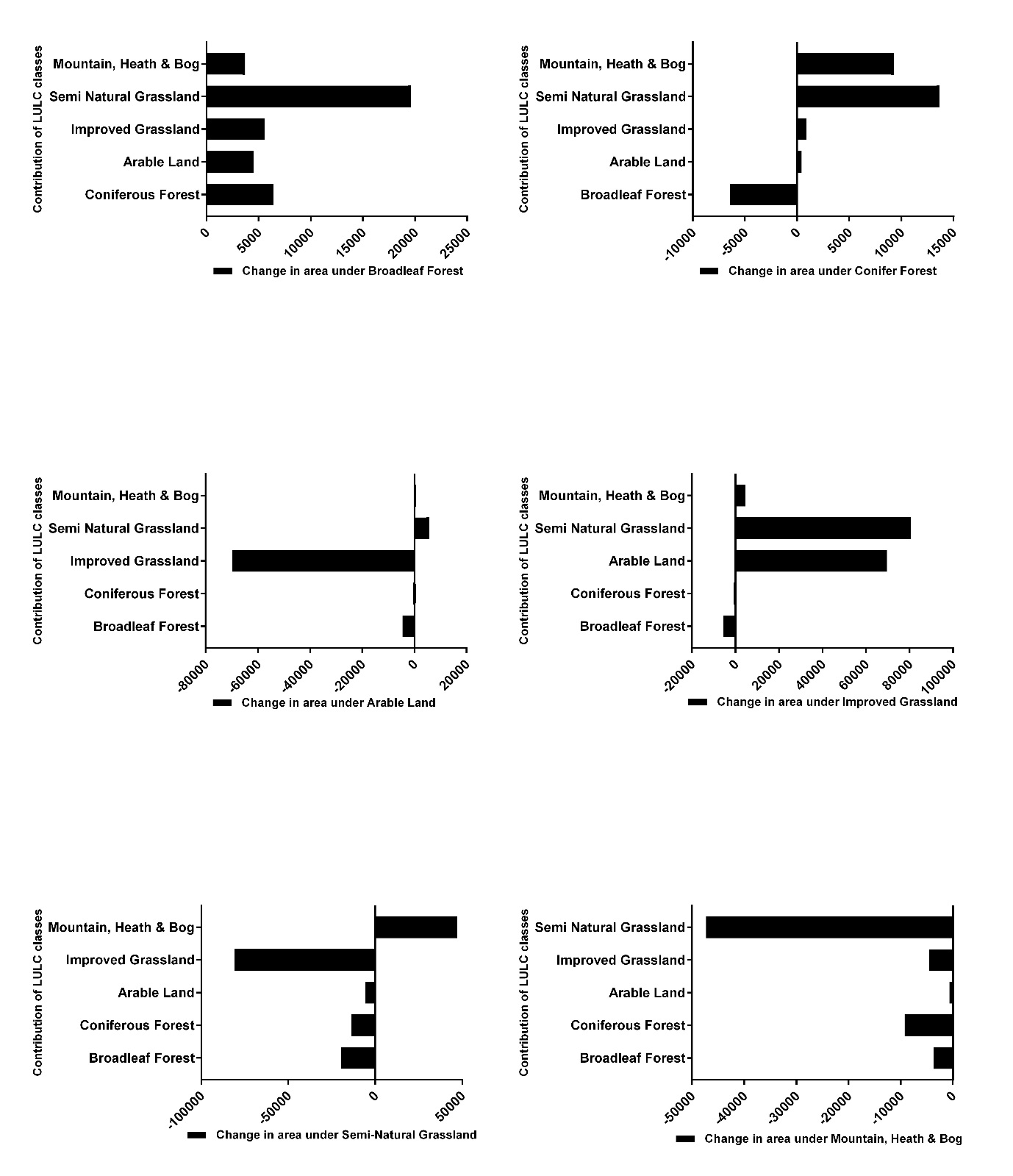
**

**Fig S1.** Contributions to net change in Broadleaf Forest, Coniferous Forests, Arable Land, Improved Grasslands, Semi Natural Grasslands and Mountain, Health & Bog in Wales, UK during 2007-2015 (hectares).


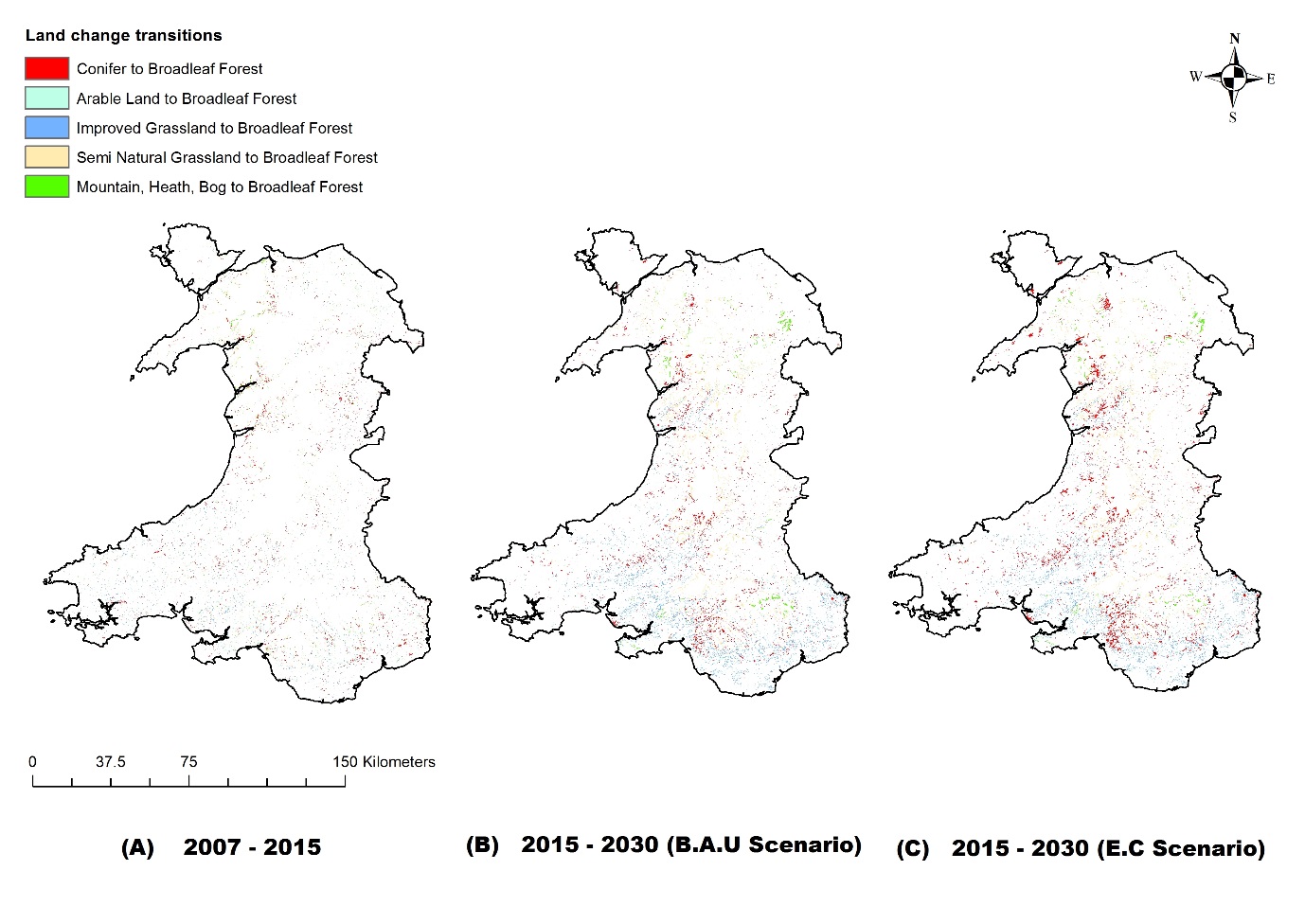


**Fig S2.** Historical land use transition maps for Wales between 2007 and 2015 (A), predicted land use transition from 2015 to 2030 under the Business-as-Usual scenario (B) and Ecosystem conservation scenario (C).


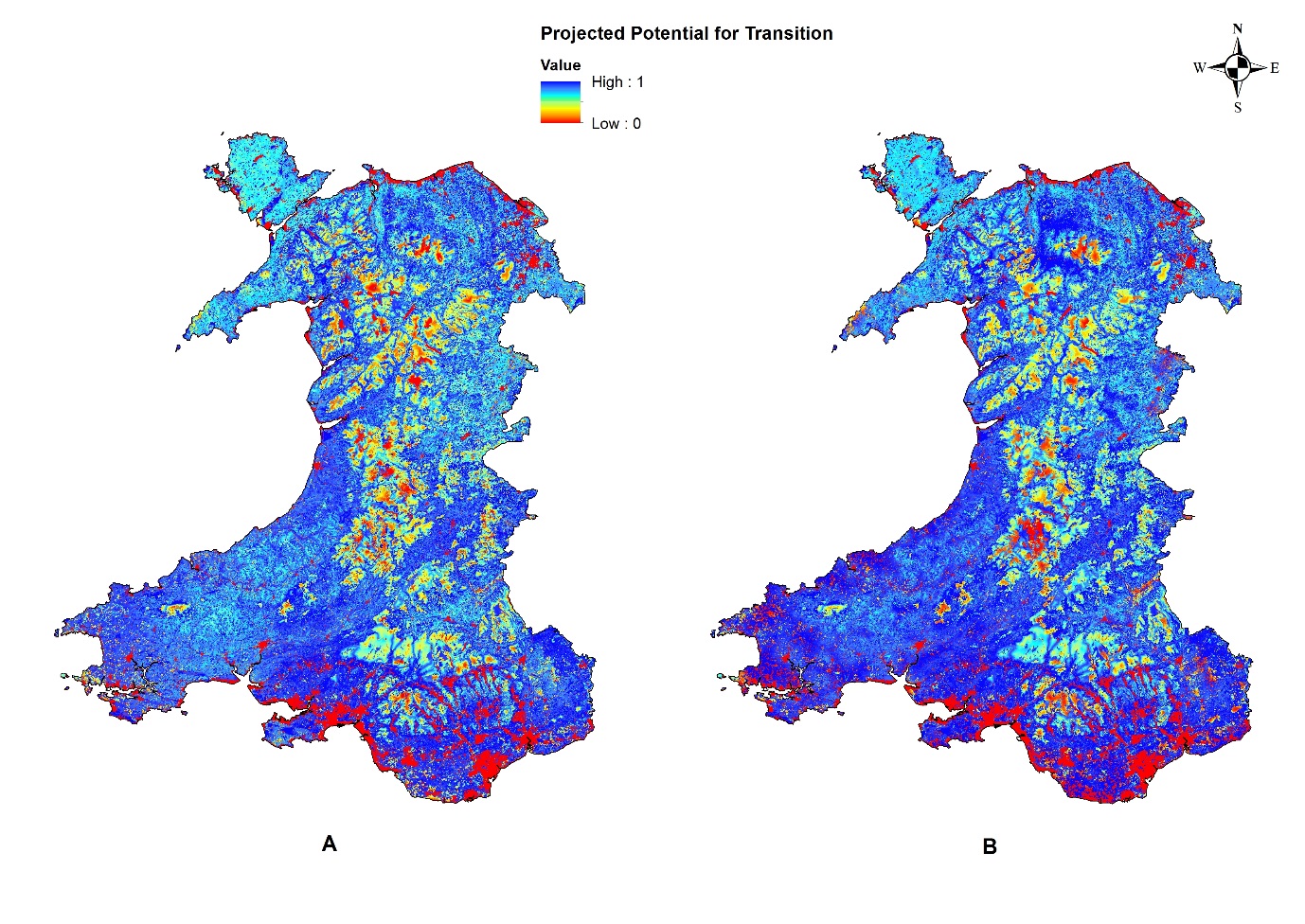


**Fig. S3.** Projected potential for transition in Wales, UK for A. Business-As-Usual Scenario & B. Ecosystem Conservation Scenario, based on the land cover transition during 2007-2015. Areas coded with numbers 0 to 1 indicate minimum to maximum potential of transition in future.
